# Preimplantation genetic testing for monogenic diseases: a Brazilian IVF centre experience

**DOI:** 10.5935/1518-0557.20180076

**Published:** 2019

**Authors:** Bianca Ferrarini Zanetti, Daniela Paes de Almeida Ferreira Braga, Matheus de Castro Azevedo, Amanda Souza Setti, Rita Cássia Sávio Figueira, Assumpto Iaconelli Jr., Edson Borges Jr.

**Affiliations:** 1 Fertility – Medical Group, São Paulo, SP – Brazil; 2 Instituto Sapientiae – Centro de Estudos e Pesquisa em Reprodução Humana Assistida, São Paulo, SP - Brazil

**Keywords:** preimplantation genetic testing (PGT), monogenic diseases, *in vitro* fertilisation (IVF), biopsy, intracytoplasmic sperm injection (ICSI)

## Abstract

**Objective:**

To describe the cases of preimplantation genetic testing for monogenic diseases (PGT-M) in fertile couples who had undergone intracytoplasmic sperm injection (ICSI) cycles in a Brazilian *in vitro* fertilisation (IVF) centre and determine whether these cases were different from those reported from the European Society of Human Reproduction and Embryology (ESHRE).

**Methods:**

This retrospective collection included data obtained from ICSI-PGT-M cycles between 2011 and 2016. The disease indication, number of biopsied embryos, biopsy stage, diagnosed and affected embryos, and cycles with embryo to transfer as well as implantation, pregnancy and miscarriage rates were analysed and compared to cycles without genetic diagnosis (PGT) and with ESHRE PGD Consortium collection XIV-XV.

**Results:**

From 5,070 cycles performed, 72 had indications for PGT-M. The most common time for biopsy was cleavage-stage; 93% of the embryos had a diagnostic result, 59.4% of which were genetically transferable, resulting in 68% of the cycles with transferred embryos, a 22.1% implantation rate, and a 28.6% pregnancy rate. No differences in clinical outcomes of cycles with PGT-M or without PGT were observed. The day of biopsy and diagnostic success as well as implantation, pregnancy and miscarriage rates were similar to ESHRE collection.

**Conclusions:**

Although the proportion of cases with PGT-M was low, its efficacy was similar to what was reported in the European collection and represents a viable alternative for families at risk of transmitting a genetic disorder to their offspring. The main difference between our and ESHRE collection were the disease indications, which reflected the admixed, multi-ethnic Brazilian population.

## INTRODUCTION

Preimplantation genetic testing for monogenic diseases (PGT-M) is a powerful tool for patients with a high risk of transmitting a genetic abnormality to their children, such as autosomal recessive, autosomal dominant and X-linked disorders (Sermon, 2002; Lee *et al*., 2017). This technology is used during intracytoplasmic sperm injection (ICSI) cycles to detect genetic traits in embryo biopsies, allowing the selection and transfer of embryos without transferring the genetic disease (Treff & Zimmerman, 2017; Sanders & Griffin, 2017). Most couples who opt for ICSI cycles with PGT-M are fertile but have already been diagnosed with a specific disorder because of a known family history or because they already have an affected child (Harper & Sengupta, 2012; Traeger-Synodinos, 2017).

The first reported successful pregnancies after human embryo biopsy for PGT-M were achieved by identifying a specific Y-chromosome sequence (Handyside *et al*., 1990). The biopsy was performed by removing one cell from cleavage-stage embryos, and the methodology used was sequence amplification by polymerase chain reaction (PCR).

Although most cycles with PGT indication perform trophectoderm biopsy nowadays (Weizman *et al*., 2018; Sullivan-Pyke & Dokras, 2018), the last European Society of Human Reproduction and Embryology (ESHRE) Preimplantation Genetic Diagnosis (PGD) Consortium collection XIV-XV showed that the most prevalent time for biopsy was still cleavage-stage, accounting for 93% of the PGT-M cycles (De Rycke *et al.,* 2017). Given that ESHRE collection is retrospective, and data presented in 2017 represent global outcomes of cycles performed from 2011-2012, it is important to highlight that the prevalence of trophectoderm biopsy must definitely increase in subsequent reports, because blastocyst biopsy has become a highly viable option, owing to the excellent survival rates of extended embryo culture and blastocyst vitrification, which gives more time for embryo diagnosis (Harper & Sengupta, 2012; Vajta & Kuwayama, 2006; Kader *et al*., 2009; Youssry *et al*., 2008). Another advantage of blastocyst biopsy is that it usually obtains between 5 and 10 cells, allowing a more reliable diagnosis than the 1 to 2 cells obtained from cleavage-stage embryo biopsy (Harton *et al*., 2011; McArthur *et al*., 2005).

Recent equipment and protocol improvements in PCR methodology consolidated it as the most reliable technique for specific amplification of a range of genetic traits, being responsible for more than 90% of PGT-M diagnoses (De Rycke *et al.,* 2015; 2017). The PCR improvements together with the increasing use of next-generation sequencing (NGS), whole genome amplification (WGA), comparative genomic hybridization (CGH) arrays and single nucleotide polymorphism (SNP) arrays have increased the PGT-M disease diagnostic spectrum and application (Briton-Jones *et al.,* 2017; Spits & Sermon, 2009; Treff *et al*., 2016; Harper *et al*., 2018; Goldman *et al*., 2016).

According to the ESHRE PGD Consortium collection XIV-XV, the most diagnosed disorders include Duchenne muscular dystrophy, Becker muscular dystrophy, fragile X syndrome, haemophilia, cystic fibrosis, thalassaemia, sickle cell anaemia, spinal muscular atrophy, myotonic dystrophy type 1 and Huntington's disease (De Rycke *et al*., 2017). Certainly, among the more than 200 indications for monogenic diseases described, the PGT-M indication in each *in vitro* fertilisation (IVF) centre depends on its incidence in the local population, which varies according to ethnicity and geographical region.

Our aim is to describe the cases of PGT-M in fertile couples who had undergone ICSI cycles in a Brazilian IVF centre and determine whether these cases were different from those reported from the ESHRE PGD Consortium collection XIV-XV.

## MATERIAL AND METHODS

### Study design

This retrospective collection study included data obtained from 5,070 cycles performed between January 2011 and December 2016 at a private university-affiliated IVF centre in Brazil. From that, 72 cycles were performed in couples who had no known history of infertility and underwent ICSI specifically for the selection of disease-free embryos. The data included information about patient history, PGT-M indication, ICSI outcomes and PGT-M analysis and results as well as implantation, pregnancy and miscarriage rates.

Written informed consent was obtained from all participants, who agreed to share their cycle outcomes for research purposes, and the local institutional review board approved the study.

### Controlled ovarian stimulation

Controlled ovarian stimulation was achieved using a daily dose of recombinant Follicle-stimulating hormone (r-FSH, Gonal-F^®^, Merck KGaA, Darmstadt, Germany) beginning on day 3 of the cycle. Pituitary blockage was performed using a Gonadotropin-releasing hormone antagonist (GnRHa, Cetrotide^®^; Merck KGaA, Darmstadt, Germany), beginning when at least one follicle ≥ 14 mm was visualised. Follicular growth was monitored using transvaginal ultrasound examination starting on day 4 of gonadotropin administration and when adequate follicular growth and serum E2 levels were observed, recombinant Human Chorionic Gonadotropin (r-hCG, Ovidrel^®^, Merck KGaA, Darmstadt, Germany) was administered to trigger the final follicular maturation. The oocytes were collected through transvaginal ultrasound ovum pick-up 35 hours after hCG administration.

### Preparation of oocytes

Retrieved oocytes were maintained in a culture medium (Global^®^ for fertilisation, LifeGlobal, Connecticut, USA) supplemented with a 10% protein supplement (LGPS, LifeGlobal, Connecticut, USA) and covered with paraffin oil (Paraffin Oil P.G., LifeGlobal, Connecticut, USA) before the removal of cumulus cells by the exposure to a medium containing hyaluronidase (80 IU/mL, LifeGlobal, Connecticut, USA). The remaining cumulus cells were mechanically removed by gently pipetting with a hand-drawn Pasteur pipette (Humagen Fertility Diagnostics, Charlottesville, USA). Oocytes that released the first polar body were considered mature and usable for ICSI.

### Intracytoplasmic sperm injection

ICSI was performed in a micro-injection dish prepared with 4-µL droplets of buffered medium (Global^®^ w/HEPES, LifeGlobal, Connecticut, USA) and covered with paraffin oil on the heated stage of an inverted microscope (37.0±0.5 ºC). Approximately 16 hours after ICSI, fertilisation was confirmed by the presence of two pronuclei and the extrusion of the second polar body. Embryos were maintained in a 50-µL drop of culture medium (Global^®^, LifeGlobal, Connecticut, USA) supplemented with a 10% protein supplement and covered with paraffin oil in a humidified atmosphere with 6% CO_2_ at 37 ºC for 3 to 5 days. Morphological embryo evaluations were performed on days 2 and 3 of development and were used as criteria for the expanded culture to blastocyst stage, together with patient characteristics and information obtained from previous cycles. All the embryos were cryopreserved after biopsy and transferred in subsequent cycles.

### Cleavage-stage embryo biopsy

Cleavage-stage embryos were biopsied on day 3 of development by placing each embryo into a 20-µL drop of Ca/Mg-free medium (LG PGD Biopsy Medium^®^, LifeGlobal, Connecticut, USA) and covered with paraffin oil. Each embryo was placed in its own drop and numbered appropriately. The microtools were fixed and aligned on an inverted microscope (Eclipse TE 300; Nikon, Tokyo, Japan) equipped with Hoffman modulation contrast. Biopsy was performed with the OCTAX Laser Shot™ (MTG Medical Technology, Germany) using laser pulse lengths of 5-8 ms (1.48 µm). After the zona pellucida opened, a single blastomere was aspirated gently and removed from the embryo.

### Blastocyst biopsy

For blastocyst biopsy, the embryos underwent assisted hatching with zona pellucida laser pulsing (OCTAX Laser Shot™; MTG Medical Technology, Germany) on day 3 of development. Only blastocysts that presented good-quality inner cell mass and trophectoderm were biopsied on day 5, 6 or 7 of development, according to its expansion grade. Each blastocyst was placed in a 20-µL drop of buffered medium (Global^®^ w/HEPES, LifeGlobal, Connecticut, USA) with 10% protein supplement and covered with paraffin oil. The hatching of the zona pellucida and trophectoderm was disposed at the 3 o’clock position, and gentle suction was applied to the blastocyst via a holding pipette (Humagen, Charlottesville, VA). A biopsy pipette (Humagen, Charlottesville, VA) was used to gently aspirate the trophectoderm into the bore of the needle. Laser pulses were used to "cut" the trophectoderm.

### Embryo diagnosis

The diagnosis was performed by PCR in an associated genetic laboratory, according to its established methodology. The genetic screening for the mutation identification was made first in the parents and/or related family, and then specific primers were designed for PCR amplification of defined regions of the embryo genome.

### Vitrification and warming

Embryos were vitrified on day 3 of development after cleavage-stage biopsy or day 5, 6 or 7 of development after blastocyst trophectoderm biopsy. Both vitrification and the warming procedures were performed using the Cryotop method (Kitazato, Minato-ku, Tokyo, Japan). Briefly, vitrification was achieved by exposure of embryos initially to the equilibration solution, followed by a 30-second exposure to the vitrification solution. Individual embryos were then picked up in an extremely small volume of vitrification solution and placed on top of a very fine polypropylene strip attached to a hard-plastic handle that was immediately submerged vertically into liquid nitrogen (Kuwayama *et al*., 2005).

For warming, the polypropylene strip was immersed directly into the thawing solution at 37ºC for 1 minute. Embryos were retrieved and transferred into the dilution solution for 3 minutes and then washed twice in the washing solution for 5 minutes each. Cleavage-stage embryos were warmed and evaluated; embryos with greater than 50% of the cells intact were considered viable and were incubated until the blastocyst stage when embryo transfer was performed. Blastocyst vitrified embryos were warmed and evaluated, and blastocysts that were not degenerated were transferred 2 to 4 hours after warming.

### Endometrial preparation

After menses, the endometrial development was followed by ultrasound examination, and the patients received 200 µg of transdermal 17β-oestradiol every 3 days (Estradot, Noven Pharmaceuticals Inc, S.A., Miami, FL, USA). Approximately 14 days after initiation of oestradiol administration, serum oestradiol levels and endometrial thickness were determined. When the endometrium showed proliferative morphology and thickness of at least 7.5 mm, 800 mg of progesterone was vaginally administered per day (Utrogestan, Farmoquímica, Rio de Janeiro, RJ, Brazil). Both 17 β-oestradiol and progesterone were administered concomitantly for 10 days after embryo transfer and were suspended in the presence of a negative β-hCG test. In the presence of a positive β-hCG test, the 17 β-oestradiol and progesterone treatments were maintained until weeks 6 and 12 of gestation, respectively.

### Clinical follow-up

A pregnancy test was performed 12 days after embryo transfer, and all women with a positive test had a transvaginal ultrasound scan after 2 weeks. A clinical pregnancy was diagnosed when the foetal heartbeat was detected.

The implantation rate was defined as the number of foetal sacs per number of embryos transferred. The pregnancy rate was measured per embryo transfer, and miscarriage was defined as pregnancy loss before 20 weeks.

### Statistical analyses

Univariate General Linear Model followed by Tukey HSD post hoc was used to compare the clinical outcomes of PGT-M cycles with the ESHRE PGD Consortium collection XIV-XV and ICSI cycles without PGT, which includes all cases of PGT-M and preimplantation genetic testing for aneuploidy (PGT-A). The results were expressed as percentages and *p* values, and the α adopted was 5%. The analysis was performed using SPSS Statistics 21 (IBM, New York, New York, USA).

## RESULTS

**Figure 1:**
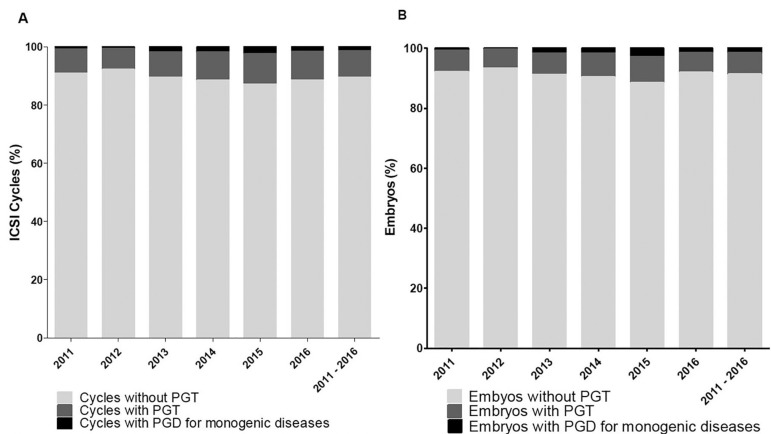
Cumulative data for each indicated group: ICSI PGT-M, ICSI PGT, and ICSI cycles without PGT. A) Proportion of cycles for each indication. B) Proportion of embryos for each indication.

Between 2011 and 2016, 5,070 ICSI cycles were performed, from which 10.4% had PGT (528/5,070). Seventy-two ICSI cycles had indication for PGT-M, which represents 13.6% of the total number of PGT cycles (72/528) and 1.4% of the total number of cycles performed (72/5,070) (Figure 1a). Regarding the number of embryos, 434 embryos were biopsied for monogenic diseases, which represented 16.7% of the 2,594 embryos biopsied for PGT and 1.5% of the total ICSI embryos obtained during the 6 years (29,798 embryos) (Figure 1b). The descriptive characteristics of PGT-M cycles are described at Table 1.

**Table 1:** General characteristics of PGT-M cycles (n=72)

	Mean±SD
Maternal age (years)	32.34±4.17
Paternal age (years)	34.47±4.13
Total FSH dose administered (IU)	2751.45±440.66
Estradiol level at hCG trigger day (pg/mL)	1729.56±896.16
Follicles (n)	16.30±8.37
Oocytes (n)	11.65±5.53
MII Oocytes (n)	8.71±4.74
Fertilisation rate (%)	84.67±15.52
Obtained embryos (n)	7.28±4.07

The most common time for biopsy was cleavage-stage (392 embryos, 90.3%). Of the embryos successfully biopsied, 93% had a diagnostic result, 59.4% of which were genetically transferable (i.e., were normal or genetic carriers). Sixty-eight percent of cycles had at least one embryo to transfer, resulting in implantation and pregnancy rates of 22.1% and 28.6%, respectively (Table 2).

**Table 2:** Outcomes of ICSI PGT-M cycles

	Cases (percentage)
PGT cycles / total ICSI cycles	528/5,070 (10.4%)
PGT-M cycles/PGT cycles	72/528 (13.6%)
PGT-M cycles/total ICSI cycles	72/5,070 (1.4%)
-PGT-M blastocyst biopsy/PGT-M cycles	7/72 (9.7%)
-PGT-M blastocyst biopsy/PGT cycles cycles	7/528 (1.3%)
-PGT-M blastocyst biopsy/total ICSI cycles	7/5,070 (0.13%)
PGT-M embryos biopsied/PGT embryos	434/2,594 (16.7%)
PGT-M embryos biopsied/total ICSI embryos	434/29,798 (1.5%)
PGT-M embryos diagnosed/biopsied	404/434 (93%)
-Normal embryos/diagnosed	91/404 (22.5%)
-Carrier embryos/diagnosed	149/404 (36.9%)
-Affected embryos diagnosed	164/404 (40.6%)
PGT-M Cycles with an embryo to transfer	49/72 (68.0%)
PGT-M Cancellation rate	23/72 (32.0%)
PGT-M Implantation rate (foetal sacs/embryos transferred)	17/77 (22.1%)
PGT-M Pregnancy rate (per embryo transfer)	14/49 (28.6%)
PGT-M Miscarriage rate	1/14 (7.1%)
PGT-M Live birth rate	13/49 (26.5%)

Compared to the last ESHRE PGD Consortium collection XIV-XV (Table 3), a higher cancellation rate (32.0% *vs.* 20.0%, *p*=0.047) was observed in our data. However, implantation rates (22.1% *vs.* 23.0%, *p*=0.733), pregnancy rates (28.6% *vs.* 26.0%, *p*=0.996) and miscarriage rates (7.1% *vs.* 10.0%, *p*=0.867) were similar.

**Table 3:** Comparison of clinical outcomes of ICSI PGT-M cycles from 2011 to 2016 with ESHRE PGD Consortium data XIV-XV

	ICSI PGT-M	ESHRE PGD XIV-XV	*p* value
Cancellation rate	32.0%	20.0%	0.047
Implantation rate	22.1%	23.0%	0.733
Pregnancy rate	28.6%	26.0%	0.996
Miscarriage rate	7.1%	11.0%	0.867

Comparing our own data on ICSI cycles without PGT in the same period (Table 4), we did not observe statistically significant differences between cancellation (33.3% *vs.* 32.0%, *p*=0.965), implantation (20.3% *vs.* 22.1%, *p*=0.931), pregnancy (33.8% *vs.* 28.6%, *p*=0.715) and miscarriage (14.7% *vs.* 7.1%, *p*=0.764) rates.

**Table 4:** Comparison of clinical outcomes of ICSI- PGT-M cycles from 2011 to 2016 with ICSI cycles without PGT from the same period.

	ICSI PGT-M	ICSI cycles without PGT	*p* value
Cancellation rate	32.0%	33.3%	0.965
Implantation rate	22.1%	20.3%	0.931
Pregnancy rate	28.6%	33.8%	0.715
Miscarriage rate	7.1%	14.7%	0.764

**Figure 2:**
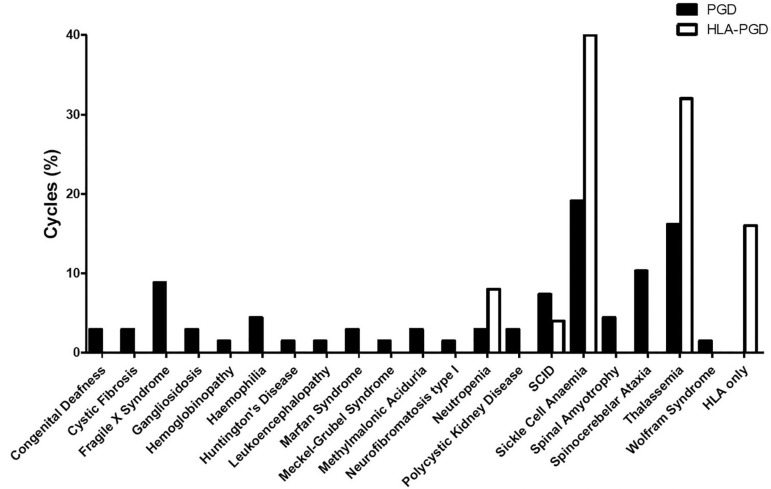
Cumulative proportion of PGT-M and HLA PGT-M cycles per monogenic disease indication, from 2011 to 2016.

Overall, the main indication for PGT-M throughout the analysed years were sickle cell anaemia (19.1%) and thalassaemia (16.2%), followed by spinocerebellar ataxia (10.3%), fragile X syndrome (8.8%) and severe combined immunodeficiency (SCID) (7.3%) (Figure 2). Other indications contributed to less than 5% of the PGT-M cycles. None of the indications were present throughout all years analysed, indicating the high heterogeneity of the patients that seek treatment at the IVF centre.

Twenty-one cycles included human leucocyte antigen (HLA) analysis together with specific disease analysis for the selection of compatible HLA-matched embryos. The cycles were performed for sickle cell anaemia (40%), thalassaemia (32%), neutropoenia (8%) and SCID (4%) (Figure 2). The cycles performed only for HLA genotyping represented 16% of the HLA- PGT-M cycles.

## DISCUSSION

PGT-M is one of the most technically challenging techniques, but it is also one of the most rewarding, since it represents a viable alternative for families at risk of transmitting a serious genetic disorder to their offspring and allows us to potentially eliminate the disease from the population. In this collection, we described the monogenic disease indications for PGT-M and its outcomes during the past 6 years in a Brazilian IVF centre. The purpose of data collection was to provide accountability, assurance of safety and efficacy for doctors and patients.

To compare our data with worldwide trends, we extract data from recently published ESHRE collection XIV-XV (De Rycke *et al*., 2017). Although ESHRE collection applies to only 71 participating centres, it is the largest and most updated database in PGT and its guidelines and best practices are applied in most IVF centres. It is important to mention that ESHRE collection refers to cycles performed worldwide in 2011-2012, while our collection refers to the period between 2011 and 2016.

The proportion of embryos biopsied for monogenic diseases was 16.7% of the PGT cycles, which is very similar to the number reported (17%) in the latest ESHRE Consortium data (Harper *et al.,* 2012; De Rycke *et al.,* 2017). For single gene defects, the use of ICSI is recommended to prevent paternal contamination from excess sperm adhered to the zona pellucida (Harton *et al.*, 2011). In this report, ICSI was used in 100% of the cases, in accordance with that reported in the ESHRE collection. Cleavage-stage embryo biopsy was most frequent (90.3%), similar to the 93% rate reported (De Rycke *et al.*, 2017). Our proportion of embryos with diagnostic results was slightly higher than that of the European report (93% *vs.* 91%). Based on Mendelian genetics, 50-75% of embryos in a single gene disorder PGT-M are expected to be unaffected by the at-risk disease (i.e., they are either normal or carriers) (Harper *et al*., 2012), and the proportion we observed in this study was in accordance to it.

The proportion of cycles with embryos to transfer was lower than that reported in the ESHRE collection (De Rycke *et al*., 2017). The subsequent cancellation rate was higher in our centre, but it was still in accordance with the cancellation rate of ICSI cycles without PGT, which shows that PGT-M in our centre can’t be correlated with an increased cancellation rate. In the same way, implantation and pregnancy rates in cycles with PGT-M or without PGT were similar. Our implantation, pregnancy and miscarriage rates were very similar to those of the ESHRE collection.

In our collection, the main PGT-M indications were sickle cell anaemia and thalassaemia, which are common syndromes in African and Mediterranean countries, respectively. In fact, the prevalence of sickle cell anaemia is higher among African descents in Brazil, and thalassaemia is higher among people of European descent. However, the real incidence of these diseases in the Brazilian population is uncertain, making it difficult to predict how many families could benefit from PGD (Lervolino *et al*., 2011; Zago & Costa, 1985).

PGT-M is now widely accepted for selecting embryos that are free of disease and histocompatible with a sibling to allow a bone marrow transplant (Harper *et al*., 2012; Renwick & Ogilvie, 2007; Kakourou *et al*., 2017). Our group was the first to describe a "saviour sibling" born after IVF and PGT-M for β thalassaemia in Brazil (Figueira *et al*., 2012). Sickle cell anaemia and thalassaemia were the most prevalent diseases in those ICSI HLA-PGT-M matching cases, which accounted for more than 70% of cases.

Despite the wide application of PGT-M, it is important to mention its disadvantages. For the patient, the most difficult part of PGT-M is that fertile couples have to undergo IVF, which is costly and potentially stressful, and it often needs to be combined with genetic and reproductive counselling (Geraedts, 2017). For the IVF centre, the concerns are the selection of the most efficient approach for each case and how to deal with cases of misdiagnosis and cycles without embryos to transfer (Wilton *et al*., 2009; Sermon, 2002). A broader scope of PGT-M technique is still limited in developing countries like Brazil, since higher priorities concerning basic health care usually take precedence over genetic diseases and birth defects among medical professionals and public health officials (Marques-de-Faria *et al*., 2004).

This report is limited by its retrospective nature and the fact that some potentially biasing covariates were not collected, such as embryo quality, number of prior attempts and number of embryos available for biopsy. Moreover, the comparison to ESHRE collection of data obtained in 2011-2012 may not reflect a direct correlation of our collection with what is now happening worldwide, and further studies comparing cycles in the same time period are needed.

In conclusion, the data we present did not differ on clinical outcomes from that of ICSI cycles without PGT. No differences in terms of day of biopsy and diagnostic success as well as implantation, pregnancy and miscarriage rates were observed in comparison to ESHRE PGD Consortium collection XIV-XV. However, the main diseases differed greatly between the collections and may reflect the admixed, multi-ethnic population of Brazil. Although the number of PGT-M cycles is still low, its efficacy is similar to what is reported worldwide and represents a viable alternative for affected families.
